# Molecular Modeling and In Vitro Studies of a Neutral Oxime as a Potential Reactivator for Acetylcholinesterase Inhibited by Paraoxon

**DOI:** 10.3390/molecules23112954

**Published:** 2018-11-12

**Authors:** Reuel L. de Paula, Joyce S. F. D. de Almeida, Samir F. A. Cavalcante, Arlan S. Gonçalves, Alessandro B. C. Simas, Tanos C. C. Franca, Martin Valis, Kamil Kuca, Eugenie Nepovimova, José M. Granjeiro

**Affiliations:** 1 National Institute of Metrology, Quality and Technology (INMETRO), Avenida Nossa Senhora das Graças 50, Duque de Caxias 25250-020, Brazil; reuel.lp@gmail.com; 2 Brazilian Army CBRN Defense Institute (IDQBRN), Avenida das Américas 28705, Rio de Janeiro 23020-470, Brazil; samir.cavalcante@eb.mil.br; 3 Laboratory of Molecular Modeling Applied to Chemical and Biological Defense, Military Institute of Engineering, Praça General Tibúrcio 80, Rio de Janeiro 22290-270, Brazil; joycesfdalmeida@gmail.com (J.S.F.D.d.A.); tanosfranca@gmail.com (T.C.C.F.); 4 Walter Mors Institute of Research on Natural Products, Federal University of Rio de Janeiro (UFRJ), CCS Bloco H Cidade Universitária, Rio de Janeiro 21941-902, Brazil; abcsimas@nppn.ufrj.br; 5 Federal Institute of Education, Science and Technology, Avenida Ministro Salgado Filho S/N, Vila Velha 29106-010, Brazil; arlansgoncalves@gmail.com; 6 Center for Basic and Applied Research, Faculty of Informatics and Management, University of Hradec Králové, Rokitanskeho 62, 50003 Hradec Králové, Czech Republic; 7 Department of Neurology, Charles University in Prague, Faculty of Medicine in Hradec Králové and University Hospital, Simkova 870, 50003 Hradec Králové, Czech Republic; Valismar@seznam.cz; 8 Department of Chemistry, Faculty of Science, University of Hradec Králové, Rokitanskeho 62, 50003 Hradec Králové, Czech Republic; evzenie.n@seznam.cz

**Keywords:** acetylcholinesterase, neutral oxime, molecular modeling, multicriteria decision making, TOPSIS-AHP, Ellman’s method

## Abstract

The present work aimed to compare the small, neutral and monoaromatic oxime, isatin-3-oxime (isatin-O), to the commercial ones, pralidoxime (2-PAM) and obidoxime, in a search for a new potential reactivator for acetylcholinesterase (AChE) inhibited by the pesticide paraoxon (AChE/POX) as well as a novel potential scaffold for further synthetic modifications. The multicriteria decision methods (MCDM) allowed the identification of the best docking poses of those molecules inside AChE/POX for further molecular dynamic (MD) studies, while Ellman’s modified method enabled in vitro inhibition and reactivation assays. In corroboration with the theoretical studies, our experimental results showed that isatin-O have a reactivation potential capable of overcoming 2-PAM at the initial moments of the assay. Despite not achieving better results than obidoxime, this molecule is promising for being an active neutral oxime with capacity of crossing the blood–brain barrier (BBB), to reactivate AChE/POX inside the central and peripheral nervous systems. Moreover, the fact that isatin-O can also act as anticonvulsant makes this molecule a possible multipotent reactivator. Besides, the MCDM method showed to be an accurate method for the selection of the best docking poses generated in the docking studies.

## 1. Introduction

The intoxication by organophosphate compounds (OPs) remains a threat to human health, with thousands of deaths every year [1]. The toxicity of these compounds is due to the inhibition of the enzyme acetylcholinesterase (AChE; EC 3.1.1.7), leading to the accumulation of the neurotransmitter acetylcholine (ACh), and subsequent over-activation of cholinergic receptors in many parts of the body. Poisoning by OPs causes physiologic consequences on the central and peripheral nervous systems (CNS and PNS). Thus, miosis, bronchorrhea, bronchoconstriction, bradycardia, emesis, skeletal muscle contraction, tachycardia, seizures, respiratory arrest and other symptoms may occur, leading to death [2].

**Figure 1:**
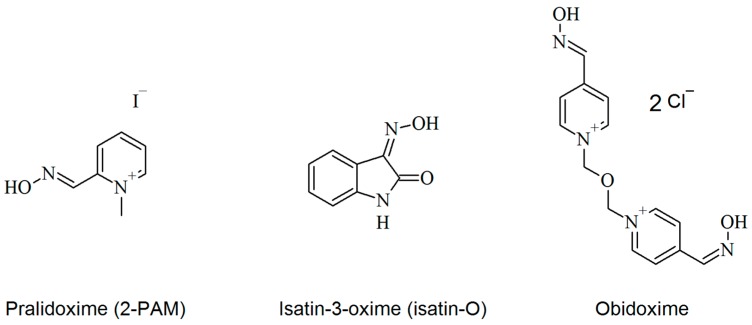
Structures 2-PAM, isatin-O and obidoxime.

The treatment for OPs poisoning is based on the administration of some drugs combined: an anticholinergic agent (e.g., atropine), an anticonvulsant drug (e.g., diazepam) and a mono- or bispyridinium AChE reactivator (e.g., pralidoxime, obidoxime, or trimedoxime) [3]. Extensive studies have investigated the mechanism of action of the oxime group in the reactivation of AChE inhibited by OPs [4,5,6,7,8,9,10,11,12,13,14,15,16,17]. Due to its structural and molecular properties, oximes are considered the most potent reactivators for AChE inhibited by OPs up to the present moment. The high affinity for AChE and nucleophilicity of these compounds allows the displacement of the OP from the catalytic site, enabling the reactivation of AChE. Pralidoxime (2-PAM) (Figure 1) is a monoquaternary oxime and one of the most employed as antidote today, although research indicates that bisquaternary oximes are more effective in the reactivation process [18]. 2-PAM is commonly used in the United States, while bispyridinium oximes, such as obidoxime (Figure 1), are used in European countries [19].

**Figure 2:**
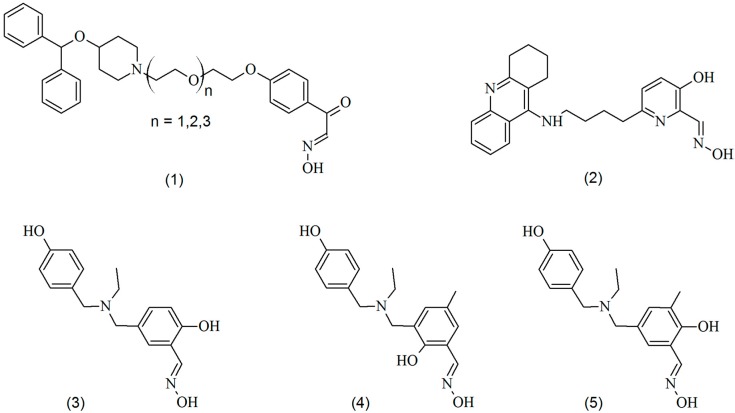
Structures of some neutral oximes reported in literature (**1** [11], **2** [18] and **3**–**5** [22]).

Despite being used commercially, quaternary oximes still present serious disadvantages. Due to their permanent charge, they poorly cross the blood–brain barrier (BBB), limiting their efficiency as AChE reactivators in the CNS and PNS [20]. Moreover, there is no universal oxime. They have highly variable efficiency depending on the nature of the OP [21]. For this reason, studies are focused on looking for oximes that could be efficient against a larger number of OPs, conjugated with an appropriate penetration in the BBB. In this sense, neutral oximes (Figure 2) have been proposed and studied by several authors [11,18,22,23,24,25].

Isatin (1*H*-indole-2,3-dione) and its derivatives have been reported in the literature with vital importance in medicinal chemistry. These molecules have presented pharmacological activities such as anticancer, antiviral, antimicrobial, antifungal, anti-inflammatory, antioxidant, analgesic, anticonvulsant, HIV reverse transcriptase inhibition, and antidiabetic [26,27,28,29,30]. Recently, they were also used to design some new compounds for the treatment of neurodegenerative diseases and new AChE inhibitors, demonstrating to also be able to interact with cholinesterases. One of the characteristics of this group of molecules is the possibility of crossing the BBB [31,32].

Among the derivatives of isatin, isatin-3-oxime (isatin-O), presented in Figure 1, was identified as an anticonvulsant [33,34]. This characteristic is essential for the treatment of OP poisoning, once current treatments require the use of the three combined drugs mentioned above (an anticonvulsant, an anticholinergic and an oxime). However, the use of this combined therapy increases the concentration of drugs in the organism, and may result in undesirable side effects. Thus, the use of a multipotent drug with more possible actions, such as anticonvulsant and AChE reactivator, could contribute to minimize this problem.

In this way, aiming to contribute for the discovery of new active neutral oximes, that could also act as anticonvulsants, and a potential scaffold for further synthetic modifications, we conducted in silico and in vitro studies of the isatin-O on AChE from *Electrophorus electricus* inhibited by the OP paraoxon (*Ee*AChE/POX), and compared our results to those of the commercial oximes 2-PAM and obidoxime. These references were chosen due to their efficacy as in vivo AChE reactivators and for being the most commonly used oximes for the treatment of OP poisoning [20]. They have also been used as standards of comparison in several published works [6,15,20,35,36,37,38,39]. Besides, the choice of these oximes allowed us to compare the results with two structurally different references, 2-PAM, which is more similar to isatin-O, and obidoxime with a larger molecular chain (see Figure 1). Another critical aspect on this choice is the known fact that bisquaternary obidoxime is a more potent reactivator for pesticide-inhibited AChE than monoquaternary 2-PAM [35].

In the in silico studies, we used docking and molecular dynamic techniques and a new approach for evaluation and selection of poses generated in the molecular docking. Due to the large number of poses produced in the molecular docking step, the process of evaluation and selection is complex. Thus, we suggest the use of decision support methods that consider multicriteria to generate broader and more reliable solutions. The use of an efficient method in this step is important because the chosen pose is typically used to represent the behavior of the molecule in the docking studies and as initial parameters for the molecular dynamic studies. Thereby, in this work, we suggest the adoption of a multicriteria decision making methods (MCDM) to select the best poses.

For the in vitro studies, we used an Ellman’s [40] modified method [41]. Since its first publication, this is the most common and relevant assay for investigating AChE activity [42,43]. The method was developed in the early 1960s [40] and it is still in use today, with some further improving modifications [41,44]. Despite some limitations eventually found, such as the lack of low detection limit and frequent background perturbations, this method has been extensively used [42] due to its simplicity, accuracy, low cost and the large number of validated and published assays.

## 2. Results and Discussion

### 2.1. Reactivation Tests

**Figure 3:**
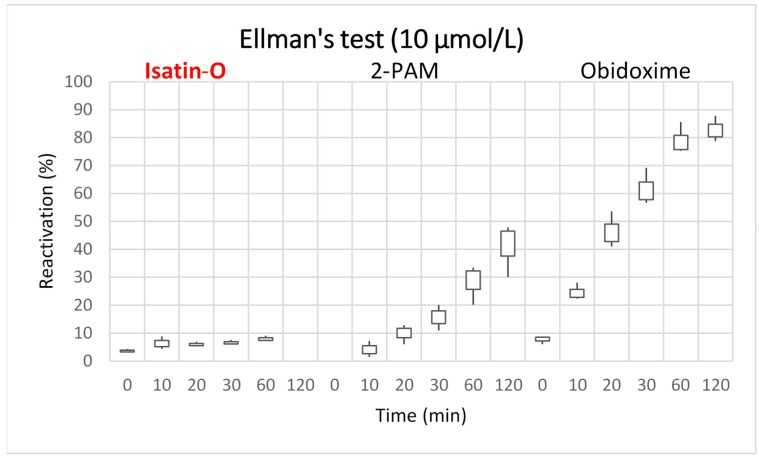
Reactivation efficacy of the oximes for *Ee*AChE/POX at 10 μmol/L.

**Figure 4:**
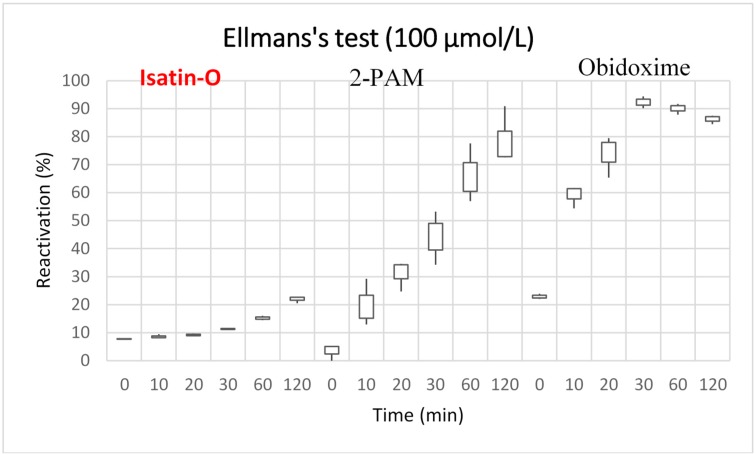
Reactivation efficacy of the oximes for *Ee*AChE/POX at 100 μmol/L.

Figure 3 and Figure 4 present the results of the reactivation tests for isatin-O, 2-PAM and obidoxime, at concentrations of 10 and 100 μmol/L respectively.

The results of Ellman’s tests [40,41] show that isatin-O can reactivate *Ee*AChE/POX at 10 and 100 μmol/L. At 10 μmol/L, it reached around 8% reactivation in the initial 10 min. At this concentration and time, 2-PAM reached close to 6% reactivation. This shows a good performance of isatin-O at short times and low concentrations, better than the results achieved by 2-PAM. Considering literature reports that 5% to 10% of reactivation is enough for the survival of the neurotoxic-intoxicated victims [45,46,47], this result points to isatin-O as a potential AChE reactivator. At 100 μmol/L, despite showing lower reactivation capacity over time, when compared to 2-PAM and obidoxime, isatin-O reached around 23% reactivation in the initial 120 min.

Among the oximes tested, the one with the highest potential for reactivation was obidoxime. This result converges with other publications [9,10,44,48]. The test performed with this oxime at 100 μmol/L indicates that the reactivation reaches a higher level after 30 min (over 90%). After this time, the reactivation rate decreases slightly. In the lower concentration range (10 μmol/L), the reactivation starts later and does not reach a maximum value before 120 min. Thus, at higher concentrations, the reactivation is more effective, allowing to reach the optimal value in a shorter time. In silico studies complemented and better explained the results of the in vitro tests and the molecular interactions present in the enzymatic reactivation.

### 2.2. Molecular Docking Studies

**Figure 5:**
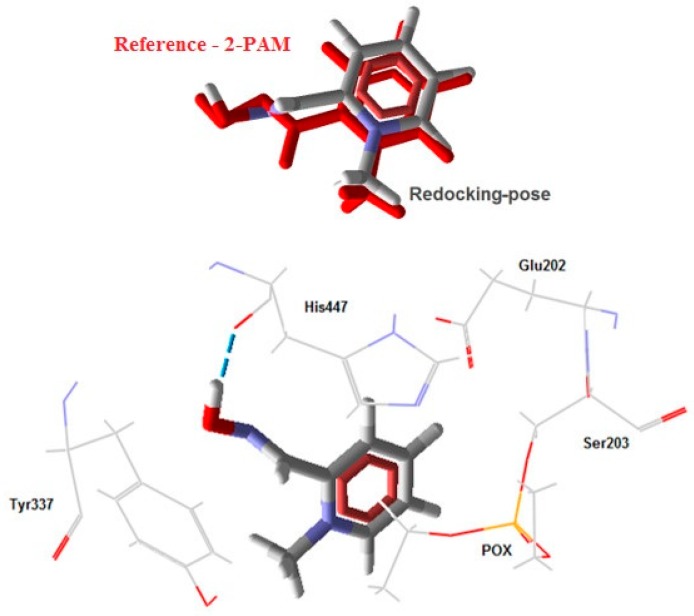
Best redocking pose.

To validate the docking protocol, redocking studies were performed using 2-PAM from the crystallographic structure as reference. The best conformation obtained (Figure 5), chosen according to the criterion of better superposition of the non-hydrogen atoms, presented random mean square deviation (RMSD) of 0.535 Å, intermolecular energy of −79.97 Kcal/mol, and hydrogen bonding energy of −2.03 Kcal/mol. Since a RMSD value under 2.0 Å is considered acceptable [48], this result validates the docking protocol used.

After validation, molecular docking studies were performed for the oximes inside *Ee*AChE/POX, and most of the results obtained corroborated the in vitro assay. Table 1 presents a summary of the docking results for the best poses selected for each oxime. The following parameters were considered: (1) distance between the oxygen atom of the oxime and the phosphorus atom of POX; (2) intermolecular energy; (3) energy of interaction related to H-bonds; (4) residues involved in H-bond interactions and, in the last column; and (5) the in vitro results of percentage of reactivation promoted by the oximes at 10 min, and at the concentration of 10 µmol/L. The choice of 10 min of reactivation time was made to evaluate the initial action of the reactivator in a possible intoxication treatment [35]. The best poses for each oxime were chosen using the MCDM [49,50,51] hybrid method: Technique for Order Preference by Similarity to Ideal Solution–Analytic Hierarchy Process (TOPSIS-AHP) [52,53,54,55,56,57]. The results are discussed in the next section. Obidoxime presented the lowest binding and H-bond interaction energies. This suggests higher affinity for the active site in comparison to 2-PAM and isatin-O. The highest energy values observed for 2-PAM point to an advantage for isatin-O over 2-PAM in terms of potential reactivation at the first moments of intoxication and corroborate the in vitro results. In addition, isatin-O showed the shortest O_(oxime)_-P_(POX)_ distance. This means that this oxime can get closer to POX than the commercial oximes, to trigger the reactivation reaction.

**Figure 6:**
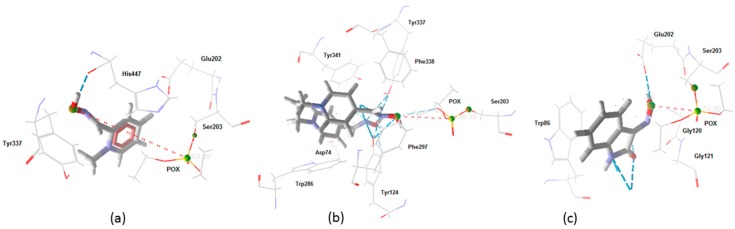
Best docking poses for: (**a**) 2-PAM; (**b**) obidoxime; and (**c**) isatin-O.

Obidoxime presented the highest number of H-bond interactions, probably due to the two oximate groups present on its structure. Results for the O_(oxime)_-P_(POX)_ distance, intermolecular and H-bond energies, and residues of interaction, point to this oxime as the best reactivator, followed by isatin-O, and finally 2-PAM. This is in corroboration with the better reactivation results presented by obidoxime, and suggest that isatin-O is a potential reactivator for AChE/POX. However, after 10 min, 2-PAM presented better reactivation results than isatin-O. This fact may occur due to the interaction of 2-PAM with one of the residues of the catalytic triad, His447, and also because 2-PAM is a positively charged molecule, a fact that favors interactions in the active site and, in consequence, the stabilization of the compound inside the enzyme. Figure 6 shows the best poses for isatin-O, obidoxime and 2-PAM. These poses were selected using the MCDM [49,50,51] hybrid method TOPSIS-AHP [52,53,54,55,56,57].

### 2.3. MCDM Method

This work adopted a hybrid multicriteria MCDM [49,50,51] hybrid method TOPSIS-AHP [52,53,54,55,56,57]. AHP method [55,56,57] was used to define the criteria weights and TOPSIS method [52,53,54] was used for the general assessment of poses from the docking studies. The methodology used in the docking calculations allowed the generation of 300 poses for each oxime studied (isatin-O, 2-PAM and obidoxime), totalizing 900 poses. This shows the importance of having an appropriate method to select the best poses, which will serve as the basis for evaluation of the interactions of these oximes in the active site, and further molecular dynamics simulations. Although the MCDM methods [49,50,51] are efficient, 300 poses generate a very large evaluation matrix, resulting in extensive and laborious calculations. Thus, to reduce the number of poses and to optimize the calculations, a screening was done. The following elimination criteria were considered in the screening: (1) positive interaction energy; (2) positive H-bond energy; (3) complete absence of interaction with active site residues; and (4) similarity. In addition, from the remaining poses, one per docking run was selected to remain in the evaluation, considering the lowest O_(oxime)_-P_(POX)_ distance, and the lowest interaction energy. As 10 docking runs were done per each oxime, at least the best 10 poses remained to be evaluated.

The evaluation steps described in the TOPSIS methodology [52,53,54] were followed. First, the performances of the poses were obtained using the docking method described. A decision matrix (DM) was constructed for each oxime, as shown in Table 2, considering the following criteria: distance O_(oxime)_-P_(POX)_, intermolecular energy, H-bond energy and interaction residues (H-bonds). Table 2 reveals the values for the criteria weights as well as when the criterion is increasing or decreasing, represented by max and min values, respectively. The poses with better performances, according to the evaluation using the hybrid MCDM method TOPSIS-AHP [52,53,54,55,56,57], are highlighted and shown in bold.

The weights of the criteria defined for the evaluation were determined using the AHP method [55,56,57]. Four specialists (authors) evaluated the importance of the criteria presented. The weight evaluation matrix was generated with the geometric mean of the evaluations of each specialist, using the aggregation of individual judgments (AIJ), which transforms individual pairwise comparison matrices (PCMs) into a PCM group from which the group priorities are then derived [58]. Table 3 presents the individual PCMs of the four specialists who participated in the evaluation of the criteria, the PCM of the group, calculated with the AIJ aggregation method [59], and the CRs of the matrices. As can be seen, all the evaluation matrices, both individual and of the group, presented CR < 0.1, a result which confirms the good quality and approves the evaluation matrices. Therefore, the values of the weights calculated with the AHP method [55,56,57] present in Table 3 could be used with reliability in the TOPSIS method [52,53,54] for evaluation of the poses.

**Figure 7:**
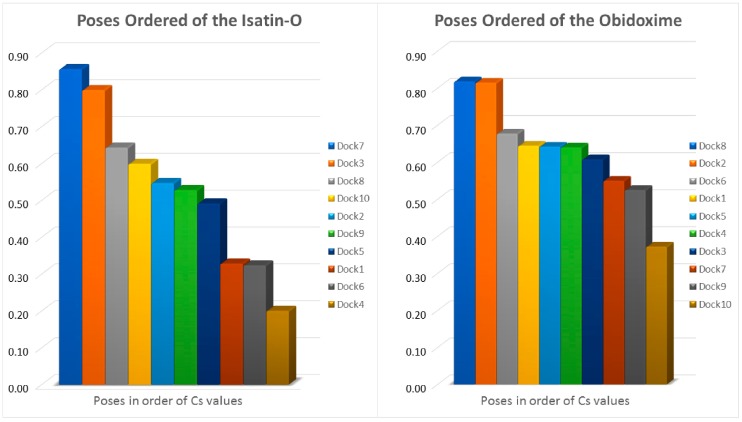
Results for the evaluation of poses through the hybrid MCDM TOPSIS-AHP method. (**Left**): Isatin-O; (**Right**): Obidoxime.

All calculations described in the methodology for the evaluation of poses were done using the TOPSIS method [52,53,54,55] with spreadsheets elaborated in excel software©. Figure 7 shows the results of the similarity coefficient calculations, for s = 2, of isatin-O and obidoxime. It was thus possible to select the best pose for each molecule, since the MCDM method employed generates, as a result, ordered poses, from the closest to the most distant solution, allowing the identification of the ideal solution. As 2-PAM was submitted to redocking, with its conformation present in the crystalline structure removed from the PDB, it was not necessary to carry out the selection of pose calculations, since, in this case, the selection met the redocking criterion. The best poses of each oxime studied were selected, as presented in Table 2 and Figure 6 and Figure 7, and then used in the further molecular dynamic simulations.

### 2.4. Molecular Dynamic Study

**Figure 8:**
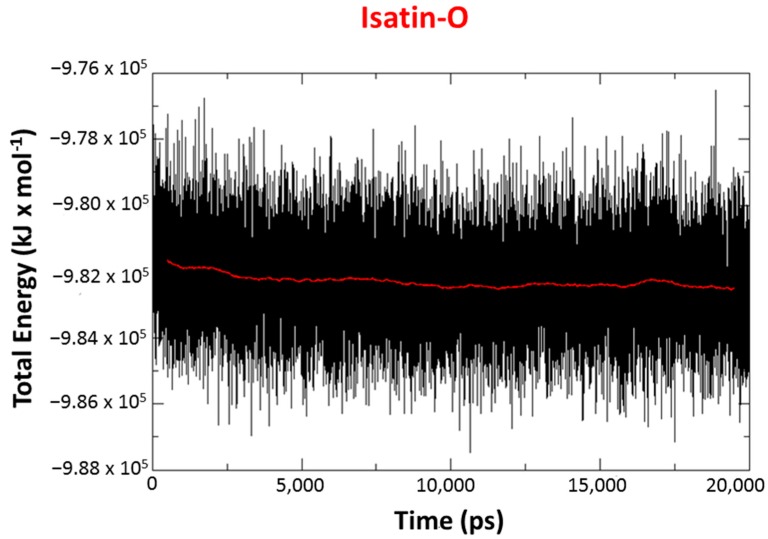
Energy plot for the complex *Ee*AChE/POX/isatin-O during 20 ns of MD simulation.

**Figure 9:**
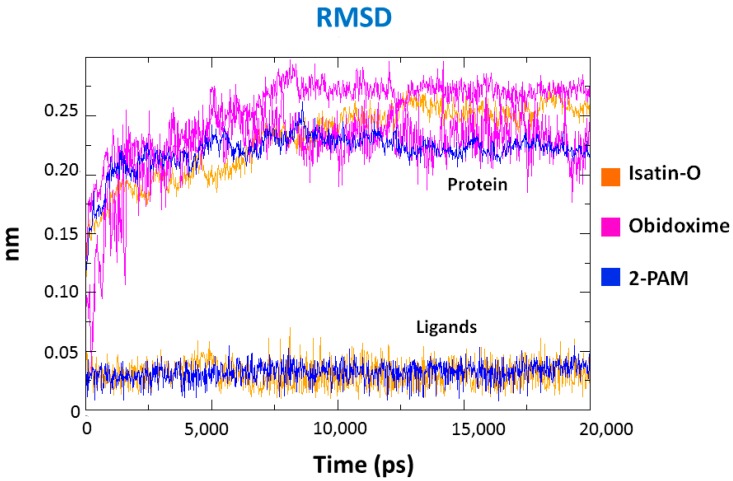
RMSD plots for the complexes *Ee*AChE/POX/ligands during 20 ns of MD simulation.

MD simulations were performed with the best poses obtained from docking studies. Results show that, for all systems, the total energy tends to stabilize after 2.5 ns of simulation, with the average values for all complexes around −9.82 × 10^5^ kJ/mol, as shown in the total energy plots for the complex *Ee*AChE-POX/isatin-O in Figure 8. In addition, the temporal RMSD plots for all systems, shown in Figure 9, confirm these results. All values obtained were below 0.3 nm (3 Å) for the protein, and below 0.05 nm (0.5 Å), for the ligands, except for obidoxime.

**Figure 10:**
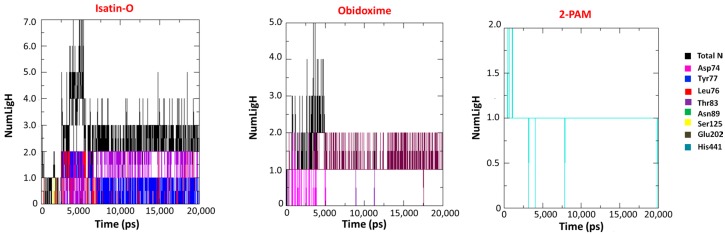
H-bond prevalence for the systems *Ee*AChE/POX/oximes.

Studies of H-bond prevalence among enzyme and ligands were performed. Results suggest that the H-bond with residue Ser125 is an important contribution for the stabilization of isatin-O inside *Ee*AChE-POX. This was observed in both docking and MD studies. Asp74 plays the same role in the complex *Ee*AChE-POX/Obidoxime. Figure 10 shows the H-bond profiles for all systems during the simulation time. It is important to notice that isatin-O presented a higher average number of H-bonds, with a higher number of interacting residues than Obidoxime and 2-PAM. Besides, isatin-O was the only compound presenting at least one H-bond during the whole simulation time. The results obtained for average number of H-bond interactions and main residues of interaction for isatin-O, 2-PAM and Obidoxime, are summarized in Table 4.

## 3. Experimental Section

### 3.1. Synthesis of the Oximes

Isatin-O was synthesized as follows [26,34]: (i) 1 mmol of the isatin was dissolved in 1 mL of water, followed by addition of 2 mmol hydroxylamine hydrochloride (NH_2_OH.HCl); (ii) the system was heated in a microwave Biotage Initiator at 120 °C for 30 min (variable power, pre-agitation of 60 s, high irradiation level); (iii) the reaction was monitored through thin layer chromatography (eluent 3:7 ethyl acetate: hexane, UV exposure and basic potassium permanganate solution); and (iv) the product was isolated through vacuum filtration, and washed with ice water, leading to the pure oxime (yield 98%). The structure and purity of the product was confirmed through spectroscopic data. ^1^H-NMR (DMSO-d_6_, δ-ppm, 400 MHz): 6.88 (d, 1H, 7.78 Hz), 7.02 (t, 1H, 7.58 Hz), 7.35 (t, 1H, 7.63 Hz), 7.95 (d, 1H, 7.49 Hz), 10.68 (s, 1H), 13.28 (s, 1H); ^13^C-NMR/DEPT-Q (DMSO-d_6_, δ-ppm, 100 MHz): 110.70, 116.41, 122.49, 127.53, 132.49, 143.07, 144.69, 164.99; GC/MS-CI (CH_4_): MW 162, [M + H]^+^ 163 *m*/*z*; MP: 234–236 °C (decomposition). Sixty-five milligrams (with 50% yield) of obidoxime dichloride were synthesized and purified as described before in the literature [46,60]. ^1^H-NMR (DMSO-d_6_, δ-ppm, 400 MHz): 6.15 (s, 4H), 8.26 (d, 4H, 6.24 Hz), 8.43 (s, 2H), 9.14 (d, 4H, 6.37 Hz), 13.09 (s, 2H); ^13^C-NMR (DMSO-d_6_, δ-ppm, 100 MHz): 86.17, 124.39, 144.89, 145.63, 150.90. 2-PAM was purchased from Sigma-Aldrich Brazil (São Paulo, Brazil).

### 3.2. Modified Ellman Method

The Ellman method was employed on the biochemical evaluation of AChE activity, being a classic method of enzymatic evaluation [40]. In this study, the method was adapted to microscale to work with small doses of OPs as well as to reduce the risks of accidental intoxication [41]. In addition, scale reduction also provided significant savings in materials and reagents. Ellman’s method were conducted in triplicate, in three different assays, by at least three different operators, measured at 24 ± 2 °C. All disposable materials and glassware in contact with OP compounds were decontaminated with aqueous solution containing 10% *w*/*v* NaOH and 10% *w*/*v* NaClO for 48 h at room temperature before correct destination and cleaning.

#### 3.2.1. Chemicals

Acetylthiocholine iodide, 5,5′-dithiobis-(2-nitrobenzoic) acid (DTNB), lyophilized *Ee*AChE (1000 U per mg protein, Type V-S, C2888), 2-PAM iodide, DMF (dry, oxygen-free sealed bottle), DMSO (biological grade, dry, oxygen-free sealed bottle), triethylamine (dry, oxygen-free sealed bottle), acetone, 4-pyridinecarboxaldehyde, sodium hydroxide (pellets), sodium phosphate monobasic hydrate, and sodium phosphate dibasic dihydrate were purchased from Sigma-Aldrich Brazil (São Paulo, Brazil). Absolute ethanol was purchased from Tedia Brazil (Rio de Janeiro, Brazil). Deuterated solvents (CDCl_3_ and DMSO-d_6_), containing tetramethylsilane as internal standard, were purchased from CIL (Tewksbury, MA, USA). Bovine Serum Albumin (BSA), purchased from Prime Alert BioDetection System, GenPrime, Spokane (Washington, DC, USA), were provided by the biological defense section of the Institute for Chemical, Biological, Radiological and Nuclear Defense (IDQBRN). Technical grade sodium hypochlorite purchased from VETEC (Rio de Janeiro, RJ, Brasil) was provided by the chemical defense decontamination team of the IDQBRN. Purified water was obtained from Millipore Milli-Q system (18.2 MΩ cm at 25 °C, Millipore Brazil, São Paulo, Brazil). Biotage Initiator 8 (Charlotte, NC, USA) was used for the synthesis of all oximes. TLC aluminum plates, coated with silica gel F254, were purchased from Merck Brazil (São Paulo, Brazil). Camag TLC-MS (AuTeC, São Paulo, Brazil) interface was used to follow up reactions. NMR spectra was obtained from Varian Unity 400 MHz and Bruker Advance 400 MHz, and referred to tetramethylsilane for ^1^H and ^13^C NMR spectra. GC-MS data were obtained from an Agilent 6890 GC system equipped with a 5975 C mass spectrometer detector. Kasvi 96-wells microplates were purchased from Kasvi Brasil (São José dos Pinhais, Paraná, Brazil), Gilson single channel were from Gilson Inc. (Middleton, WI, USA) and Eppendorf 8-channel were from Eppendorf Brasil (São Paulo, Brazil).

#### 3.2.2. Inhibition Assay

In a 96-well microplate (final volume per well of 200 μL) the following was added per well: 70 μL AChE 2.14 U/mL (0.75 U/mL per well); 80 μL DTNB 0.4 mg/mL; 20 μL PBS; and 10 μL POX 200 μmol/L (11 μmol/L per well, positive control, absorbance L_i_). After 10 min of incubation, 20 μL of 1 mmol/L ATCI were added and, after additional 10 min, absorbance was read at 412 nm. PBS (10 μL) was used as negative control (absorbance L_0_). AChE inhibition was calculated using Equation (1):

%I = [(L_0_ − L_i_)/L_0_] × 100 (1)

where %I is the inhibition percentage, L_0_ is the absorbance without inhibitor, and L_i_ is the absorbance with inhibitor.

#### 3.2.3. Reactivation Assay

In a 96-well microplate (final volume per well of 200 μL) the following was added per well: 70 μL AChE 2.14 U/mL (0.75 U/mL per well); 80 μL DTNB 0.4 mg/mL; and 10 μL POX 200 μmol/L (11 μmol/L per well, positive control). After 10 min of incubation, 20 μL of standard antidote or test molecule were added, followed by further 10 min incubation. Then, 20 μL of 1 mmol/L ATCI were added and the absorbance (L_r_) was read after 10 min. AChE reactivation was calculated using Equation (2):

%R = [(Lr − Li)/(L_0_ − Li)] × 100 (2)

where %R is the reactivation percent, L_0_ is the absorbance without inhibitor, L_i_ is the absorbance with inhibitor, and L_r_ is the absorbance after addition of reactivator.

### 3.3. Molecular Modeling Studies

Docking and MD studies were carried out to verify the binding modes of isatin-O inside inhibited *Ee*AChE and compared its reactivation potential to the commercial oximes 2-PAM and Obidoxime. The model of *Ee*AChE inhibited by POX was used in this work to be as close as possible to the experimental in vitro test. It was constructed using human AChE (*Hss*AChE) inhibited by POX and complexed with 2-PAM (PDB code: 5HFA) as template. The primary (FASTA) sequences of *Hss*AChE and *Ee*AChE (PDB code: 1C2O) were aligned and the different residues mutated using the software spdbviewer [61] to obtain the model of *Ee*AChE inhibited by POX, and complexed with 2-PAM. Target and template presented 89% of homological identity and 100% of the active site residues conserved. The model was further validated using the server PDBSum (www.ebi.ac.uk/pdbsum). The 3D structures of more stable and active conformations of each oxime (2-PAM [62], obidoxime [63] and isatin-O [34]) were constructed through the program PC Spartan 08^®^ [64] and their partial atomic charges calculated through the RM1 (Recife Model 1) semi-empirical method [65].

#### 3.3.1. Molecular Docking

The software Molegro Virtual Docker (MVD)^®^ [66] was used to perform docking studies through the algorithm MolDock Score, an adaptation of the algorithm Differential Evolution (DE) [67]. The oximes were docked in the model after a redocking procedure to validate the methodology. The RMSD was calculated using 2-PAM as reference. The binding site was limited to a sphere with a radius of 11 Å and residues within a 10 Å radius were considered flexible. Due to the stochastic nature of the docking algorithm, about 10 runs were done for each compound, with 30 configurations (poses) returned for evaluation. The best pose of each compound was selected according to the following criteria: distance between the P atom of POX and the O atom of the oxime, interaction energy between the oxime and inhibited *Ee*AChE, energy involved on hydrogen bonds and total number of AChE residues interacting with the oxime. To select the best poses of the docking study, multicriteria decision methods (MCDM) [49,50,51] were employed. The pairwise comparison method (AHP) [55,56,57] was used to calculate the criteria weights and TOPSIS [52,53,54] was used for ranking the best poses.

#### 3.3.2. Molecular Dynamics

The poses chosen from the docking studies were parameterized for the OPLS/AA forcefield available in the GROMACS 5.1.4 program [68], and the parameters and topologies were obtained from the software AnteChamber PYthon Parcer InterfacE (ACPYPE) [69]. The complexes *Ee*AChE-POX/oxime were simulated using GROMACS 5.1.4 [68] package in a cubic box (941.59 nm^3^) containing approximated 28,522 spc216 water molecules with periodic boundary conditions. The minimization steps were steepest descent with position restrained (PR) of ligands and protein, with a convergence criterion of 100.00 Kcal/mol.Å, steepest descent without PR to flexibilize the system, conjugate gradients (CG), and L-BFGS (limited-memory Broyden–Fletcher–Goldfarb–Shanno [24]), until a minimum of energy of 1.00 Kcal/mol.Å. After that, two steps of equilibration were done. The first one under constant number of particles, volume and temperature (NVT) and the second under constant number of particles, pressure and temperature (NPT). The minimized complexes were submitted to MD simulations in two parts. First, 500 ps of MD were done at 310 K, with PR for the entire system, except the water molecules, to ensure a balance of the solvent molecules around the residues of the enzyme. After, 20,000 ps of MD were done at 310 K without any restriction, using 2 fs of integration time and a cut-off of 10 Å for long-distance interactions. Counter ions were added to neutralize the whole systems. The trajectories generated after the optimization and MD steps were visualized on VMD [70] software. Plots of total energy, distance, variation of RMSD and H-bonds formed during the MD simulation were generated on the Grace program (Version 5.1.25, http://plasma-gate.weizmann.ac.il/Grace/). Pictures of MD frames during the MD simulations were generated in the PyMOL program [71].

### 3.4. MCDM Method

The MCDM method employed for the choice of the best docking poses is presented in the Supplementary Materials [49,50,51,52,53,54,55,56,57,58,59,72,73].

## 4. Conclusions

In this work, molecular modeling, MCDM methods, and biochemical tests were performed to evaluate the neutral oxime isatin-O as a potential reactivator or scaffold for AChE/POX. Two classical oximes, 2-PAM and obidoxime were selected to serve as references in the studies. Docking and MD studies pointed to isatin-O as a potential reactivator of AChE/POX. Both methods showed isatin-O with higher reactivation potential than 2-PAM, although it did not exceed the obidoxime results. These data suggest the convergence and increasing confidence in the results.

In the same direction, Ellman’s tests also pointed to isatin-O as a potential reactivator of AChE/POX. This neutral molecule achieved a reactivation of about 23% after 120 min at 100 μmol/L and close to 10% at initial instants, demonstrating activity. At lower concentration, 10 μmol/L, it reached approximately 10% reactivation from the initial instants of the assay and remained stable during the time. As reported in the literature [45,46,47], these percentages are sufficient to keep a poisoning victim alive. Comparing with traditional reference oximes, in the initial minutes of the test, the results of isatin-O slightly exceeds 2-PAM, being overcome by obidoxime. However, the percentage of reactivation of isatin-O did not exceed the results of the two classical oximes in longer times.

Although not as efficient as obidoxime, isatin-O and possible derivatives were expected to exhibit higher capacity for BBB penetration and demonstrate promising in vivo reactivation ability as a result of their nonquaternary structures [23]. Moreover, they can act as anticonvulsants, making them possible multipotent reactivators. These results may indicate a starting point to a novel potential scaffold for further synthetic modifications and development of more efficient centrally acting reactivators for OP poisoning.

Regarding the adopted poses assessment methodology in the docking studies, the use of the hybrid MCDM method TOPSIS-AHP [52,53,54,55,56,57] contributed to improving the molecular modeling techniques. The use of this method is unprecedented in this area of study. Its application showed to be advantageous related to the conventional forms of selection of the best poses generated in the docking studies, since it considers multiple criteria of selection simultaneously and presents at the end the ordering of the best poses.

The paired comparison AHP method was used at the stage where the TOPSIS method is less efficient at the evaluation of criteria weights. Paired comparison using the Saaty scale of the AHP method revealed to be proper for the evaluation of the criteria weights. The TOPSIS method, in its turn, was used at the stage where it is most efficient, in the general evaluation of the decision matrix. Therefore, the hybrid method adopted used the strengths of each technique, generating an adequate evaluation and selection of the poses obtained from docking studies.

## Figures and Tables

**Table 1:** Docking results for the oximes inside *Ee*AChE/POX.

Oxime	Distance O(oxime)-P(POX) (Å)	Intermolecular Energy (kcal/mol)	H-Bond Energy (kcal/mol)	Interaction Residues	% Reactivation (Mean in 10 min—Conc. 10 µmol/L)
**Isatin-O**	4.03	−75.22	−1.60	Ser125	6.37
**Obidoxime**	4.60	−122.48	−11.54	Ser203 Tyr124 Tyr337 Asp74	24.55
**2-PAM**	8.14	−87.18	−2.03	His447	4.13

**Table 2:** Decision matrix (isatin-O and obidoxime). The poses with better performances are shown in bold.

Criterion	Distance O-P (Å)	Intermolecular Energy (kcal/mol)	H-Bond Energy (kcal/mol)	Interaction Residues
*Max/Min*	Min	Min	Min	Max
*Weight*	0.633	0.228	0.044	0.095
***Isatin-O***				
*Dock1*	5.19	−81.13	−4.92	2
*Dock2*	4.50	−94.18	−2.65	2
*Dock3*	3.75	−84.51	−0.6	1
*Dock4*	5.76	−81.33	−7.76	3
*Dock5*	4.68	−93.39	−2.53	3
*Dock6*	5.44	−71.62	−2.25	2
***Dock7***	**3.76**	**−76.95**	**−2.18**	**1**
*Dock8*	4.50	−78.03	−0.96	3
*Dock9*	4.60	−92.04	−3.77	3
*Dock10*	4.50	−85.00	−0.24	2
***Obidoxime***				
*Dock1*	5.28	−123.99	−8.78	4
*Dock2*	4.62	−125.10	−7.71	3
*Dock3*	5.42	−103.54	−5.33	2
*Dock4*	5.3	−111.45	−11.7	3
*Dock5*	5.29	−122.32	−5.20	3
*Dock6*	5.15	−122.76	−8.67	4
*Dock7*	5.3	−118.75	−7.22	3
***Dock8***	**4.6**	**−122.48**	**−11.54**	**4**
*Dock9*	5.28	−121.38	−7.73	3
*Dock10*	5.12	−111.4	−10.23	2

**Table 3:** Pairwise Comparison Matrices (PCMs).

**DM1**	Crit 1	Crit 2	Crit 3	Crit 4	**DM2**	Crit 1	Crit 2	Crit 3	Crit 4
Crit 1	1	5	9	7	Crit 1	1	4	9	7
Crit 2	1/5	1	5	4	Crit 2	1/4	1	7	4
Crit 3	1/9	1/5	1	1/3	Crit 3	1/9	1/7	1	1/4
Crit 4	1/7	1/4	3	1	Crit 4	1/7	1/4	4	1
CR = 0.09					CR = 0.09				
**DM3**	Crit 1	Crit 2	Crit 3	Crit 4	**DM4**	Crit 1	Crit 2	Crit 3	Crit 4
Crit 1	1	5	9	7	Crit 1	1	4	8	6
Crit 2	1/5	1	5	3	Crit 2	1/4	1	5	4
Crit 3	1/9	1/5	1	¼	Crit 3	1/8	1/5	1	1/3
Crit 4	1/7	1/3	4	1	Crit 4	1/6	1/4	3	1
CR = 0.08					CR = 0.08				
**GrPCM**	Crit 1	Crit 2	Crit 3	Crit 4	**W_i_ = Weights**				
Crit 1	1	4.47	8.74	6.74	**0.633**				
Crit 2	0.22	1	5.44	3.72	**0.228**				
Crit 3	0.11	0.18	1	0.29	**0.044**				
Crit 4	0.15	0.27	3.46	1	**0.095**				
CR = 0.083									

Crit 1, Distance O_(oxime)_-P_(POX)_; Crit 2, Intermolecular energy; Crit 3, H-bond energy; Crit 4, Interaction residues; DM, decision maker/expert; GrPCM, Group pairwise comparison matrix.

**Table 4:** Summary of MD results for all complexes *Ee*AChE/POX/ligands. The interacting residues observed also in docking studies are shown in red.

Oxime	Average H-Bond Number	Interaction Residues
**Isatin-O**	3	Leu76
		Tyr77
		Thr83
		Asn89
		**Ser125**
**Obidoxime**	2	**Asp74**
		Thr83
		Asn89
		Glu202
**2-PAM**	1	His441

## Supplementary Materials

The supplementary materials are available online, MCDM method.
