# Supplementary material for: Molecular Modeling and In Vitro Studies of a Neutral Oxime as a Potential Reactivator for Acetylcholinesterase Inhibited by Paraoxon
Source: Molecules. 2018 Nov 12;23(11):2954. doi: 10.3390/molecules23112954 (PMC6278417; doi:10.3390/molecules23112954)

molecules

Article

Molecular modeling and *in vitro* studies of a neutral oxime as a potential reactivator for acetylcholinesterase inhibited by paraoxon

Reuel L. de Paula [1,2], Joyce S. F. D. de Almeida [3], Samir F. A. Cavalcante [2,4], Arlan S. Gonçalves [5], Alessandro B. C. Simas [4], Tanos C. C. Franca [3,6], Martin Valis [7], Kamil Kuca [8,*], Eugenie Nepovimova [8] and José M. Granjeiro [1]

1 National Institute of Metrology, Quality and Technology (INMETRO), Avenida Nossa Senhora das Graças 50, Duque de Caxias 25250-020, Brazil; reuel.lp@gmail.com (R.L.d.P.); jmgranjeiro@gmail.com (J.M.G.).
2 IDQBRN (Brazilian Army CBRN Defense Institute), Avenida das Américas 28705, Rio de Janeiro 23020-470, Brazil; reuel.lp@gmail.com (R.L.d.P.); samir.cavalcante@eb.mil.br (S.F.A.C.).
3 Laboratory of Molecular Modeling Applied to Chemical and Biological Defense, Military Institute of Engineering, Praça General Tibúrcio 80, Rio de Janeiro 22290-270, Brazil; joycesfdalmeida@gmail.com (J.S.F.D.d.A.); tanosfranca@gmail.com (T.C.C.F.).
4 Walter Mors Institute of Research on Natural Products, Federal University of Rio de Janeiro (UFRJ), CCS Bloco H Cidade Universitária, Rio de Janeiro 21941-902, Brazil; samir.cavalcante@eb.mil.br (S.F.A.C.); abcsimas@nppn.ufrj.br (A.B.C.S.).
5 Federal Institute of Education, Science and Technology, Avenida Ministro Salgado Filho S/Nº, Vila Velha 29106-010, Brazil; arlansgoncalves@gmail.com (A.S.G.).
6 Center for Basic and Applied Research, Faculty of Informatics and Management, University of Hradec Králové, Rokitanskeho 62, 50003 Hradec Králové, Czech Republic; tanosfranca@gmail.com (T.C.C.F.).
7 Department of Neurology, Charles University in Prague, Faculty of Medicine in Hradec Králové and University Hospital, Simkova 870, 50003 Hradec Králové, Czech Republic. Valismar@seznam.cz (M.V).
8 Department of Chemistry, Faculty of Science, University of Hradec Králové, Rokitanskeho 62, 50003 Hradec Králové, Czech Republic; evzenie.n@seznam.cz (E.N).

* Correspondence: jmgranjeiro@gmail.com (J.M.G.); kamil.kuca@uhk.cz (K.K.); Tel.: +55-212-679-9834 (J.M.G.); +420-603-289-166 (K.K.)

Received: 28 September 2018; Accepted: 9 November 2018; Published: date

MCDM method

MCDM is a subdivision of operations research that is related with designing mathematical and computational tools [33,34]. This study area was developed to solve real decision problems and works satisfactorily in many application areas such as: health, safety and environment management; design, engineering and manufacturing systems; business and marketing management; human resources management; chemical engineering; water resources management; and others [35]. TOPSIS was proposed by Hwang and Yoon [36] and has been extensively applied in the past decades with suitable results [35]. This method is used because it is rational and comprehensible, the process is simple, and it is organized by an algorithm. It can search for the best alternatives through simple mathematical operations where the process of calculation considers the values of the weights of each criterion and if the criterion is a cost or a profit [37]. The main idea came from the concept of the compromise solution to choose the best alternative nearest to the positive ideal solution (PIS), and farthest from the negative ideal solution (NIS). The method identifies solutions in a finite number of

alternatives based on minimization of the distance from PIS and maximization of the distance from NIS simultaneously. According to Olson [38], this procedure uses the Euclidean distance technique (norm, square root of the sum of the square of the distances, the vector that separates an alternative solution ideal or not ideal). The computational steps of TOPSIS [36-38] are:

Step 1 – To obtain the performance of the alternatives x in relation to the k criteria. Usually the initial measures are normalized in order to obtain the same scale of value;

Step 2 – Development of the weights (W) for each criterion. In this work, the weight was calculated with the AHP method [39];

Step 3 – Identification of the PIS (extreme positive performance in each criterion) and the NIS (negative extreme performance in each criterion);

Step 4 – Determination of distances (d) for PIS and NIS in each criterion, as presented in Equations 4 and 5, respectively.

$$d_p^{PIS} = \left\{\sum_{j=1}^{J} W_j^p \left[\frac{f_j^* - f_j(x)}{f_j^* - f_j^-}\right]^p + \sum_{i=1}^{I} W_i^p \left[\frac{f_i(x) - f_i^*}{f_i^- - f_i^*}\right]^p\right\}^{1/p} \quad (4)$$

$$d_p^{NIS} = \left\{\sum_{j=1}^{J} W_j^p \left[\frac{f_j(x) - f_j^-}{f_j^* - f_j^-}\right]^p + \sum_{i=1}^{I} W_i^p \left[\frac{f_i^- - f_i(x)}{f_i^- - f_i^*}\right]^p\right\}^{1/p} \quad (5)$$

where: f_j^*= ideal solution for the crescent criterion;

f_i^*= ideal solution for the decrescent criterion;

f_j^-= non-ideal solution for the crescent criterion;

f_i^-= non-ideal solution for the decrescent criterion;

$f_j(x) =$ value obtained by alternative x for the crescent criterion;

$f_i(x) =$ value obtained by alternative x for the decrescent criterion;

p = parameter for checking the sensitivity, where $1 \leq p \leq \infty$;

W_j = weight assigned to the jth criterion;

W_i = weight assigned to the ith criterion;

J = total number of criteria with crescent behavior;

I = total number of criteria with decrescent behavior.

Step 5 – For each alternative, the coefficient of similarity (C), is calculated as shown in Equation 6. This coefficient represents how much the alternative in question approaches the ideal positive solution, and varies between zero and one.

$$C_s^* = \frac{d_p^{NIS}}{d_p^{NIS} + d_p^{PIS}} \quad (6)$$

Where: d_p^{PIS}= distance from the PIS to the alternative x;

d_p^{NIS}= distance from the NIS to the alternative x;

p = index of importance with respect to maximum deviation (p = 1,2 ... ∞)

$C_s^* =$ coefficient of similarity (with s = p).

Step 6 – Ordering the alternatives with the values of C, from the largest to the smallest.

One of the disadvantages of TOPSIS [36-38] is on the weights calculation. In order to overcome this deficiency, we propose to use the AHP method [39-41] to accomplish this task. In this method, the judgment of importance for each criterion is performed by paired comparison, using the

Saaty scale [40], which consists on verbal judgments ranging from equal to extreme importance (equal, moderately more, strongly more, very strongly more, extremely more), corresponding to the numerical judgments (1,3,5,7,9) and compromises between these values. The evaluation of the weights for the criteria counted on the participation of more than one decision maker. In this scenario, with multiple decision makers, conventionally are applied methods to obtain group priorities. The most widely group employed in AHP is the aggregation of individual judgements (AIJ). In this case a judgement matrix is constructed for the group. Each entry in this reciprocal matrix of pairwise comparisons (PCM) is obtained as the weighted geometric mean of individual judgments, and the priorities for the alternatives compared are calculated on this basis [43,55]. The AHP method has the advantages of producing more precise results and verifying consistency of judgments. The computational steps of AHP method in this work are the following:

Step 1 – Build PCM (employ pairwise comparison by using a nine-point scale of Saaty). The results of the comparisons are presented with an n x n PCM:

$$A = \begin{pmatrix} 1 & a_{12} & \dots & a_{1n} \\ \frac{1}{a_{21}} & 1 & \dots & a_{2n} \\ \dots & \dots & \dots & \dots \\ \frac{1}{a_{n1}} & \frac{1}{a_{n2}} & \dots & 1 \end{pmatrix} \quad (7)$$

The following conditions must be met:

$a_{ij} = \alpha$

$a_{ji} = {}^{1}/_{\alpha}$

$a_{ii} = 1$

where:

- a_{ij} = pairwise comparison between criteria i and j; and
- α = value of importance intensity.

The values on the left and right sides of the diagonal matrix represent the strength of the relative importance degree of the *i*th element compared to the *j*th element.

Step 2 – Resolution of matrix "A", which denote the importance degree for the *i*th criterion (W_i). Equation 8 expresses the normalization of the geometric mean, used in this work to determine W_i.

$$W_i = \frac{\left(\prod_{j=1}^{n} a_{ij}\right)^{1/n}}{\sum_{i=1}^{n}\left(\prod_{j=1}^{n} a_{ij}\right)^{1/n}}, \quad i,j = 1,2,\dots,n \quad (8)$$

Step 3 – Calculate the consistency of the evaluations of criterions, denominated consistency ratio (CR), which describe and reflect the quality of a PCM. In this work CR was calculated using the equation 9 [56]. The CR allows to evaluate the degree of violation of proportionality and transitivity of the judgments, evidencing the need or not to acquire more accurate information about the criteria used or perform new judgments. For Saaty [40], the consistency ratio should not have a value greater

than 0.1. If this happens the evaluator should review his judgments. Thus, the evaluations produced with CR ≤ 0.1 have good quality.

$$CR = \frac{\lambda_{max} - n}{2.7699n - 4.3513 - n} \quad (9)$$

where: λ_{max} is the largest or principal eigenvalue of A.

The poses selected with the hybrid MCDM method TOPSIS-AHP [36-38,39-41] proposed in this paper were used at of the molecular dynamics (MD) simulations.

NMR spectra supporting identification of isatin-O

¹H NMR of isatin-O

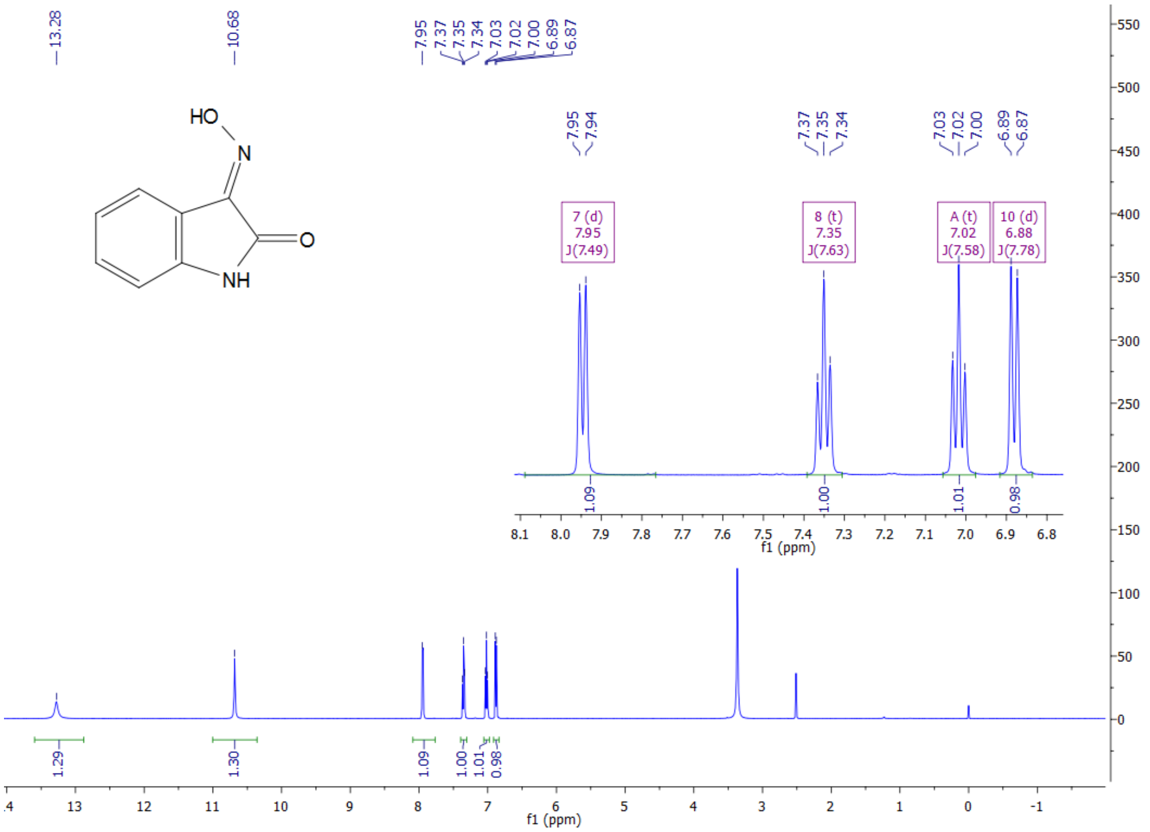

¹³C NMR of isatin-O

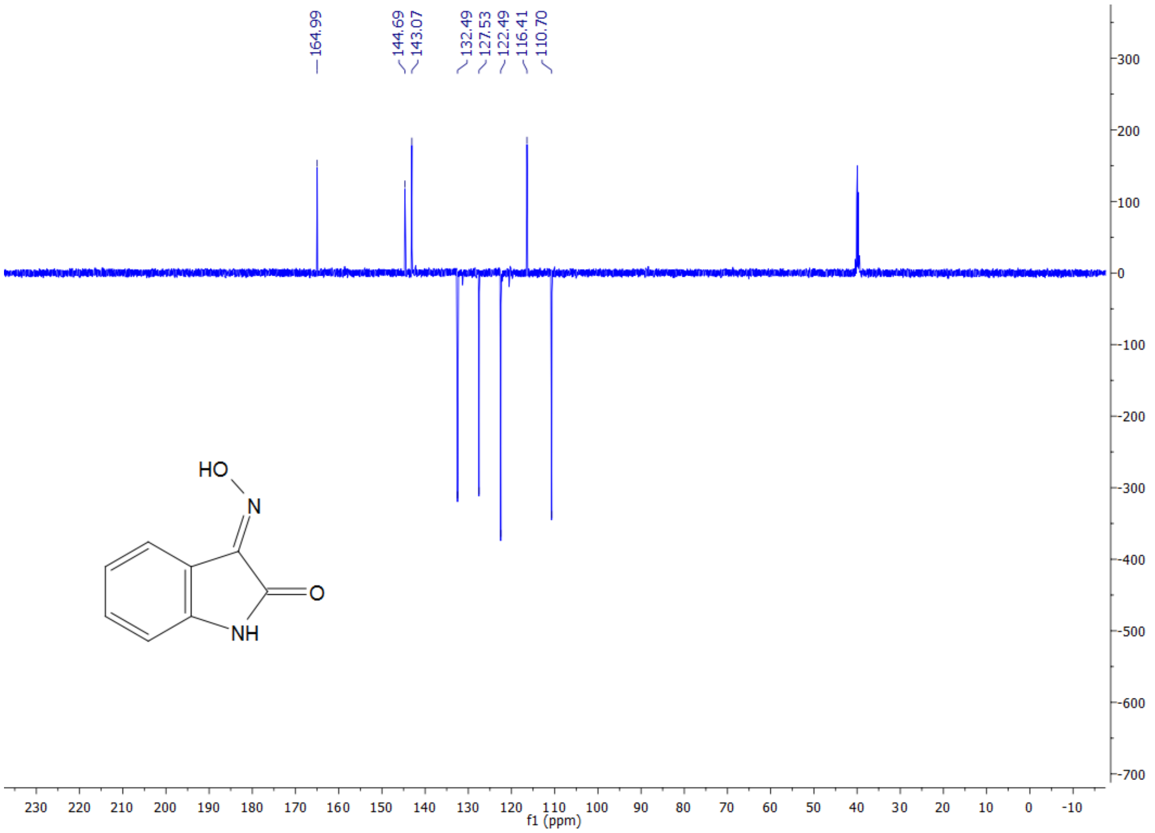

CI (methane positive) mass spectra supporting identification of Isatin-O (MW 162)

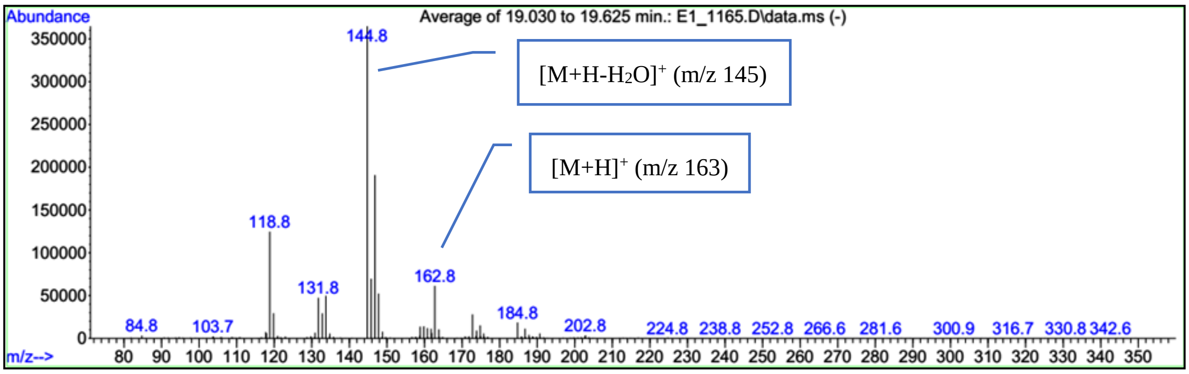

NMR spectra supporting identification of obidoxime

^{1}H NMR of obidoxime

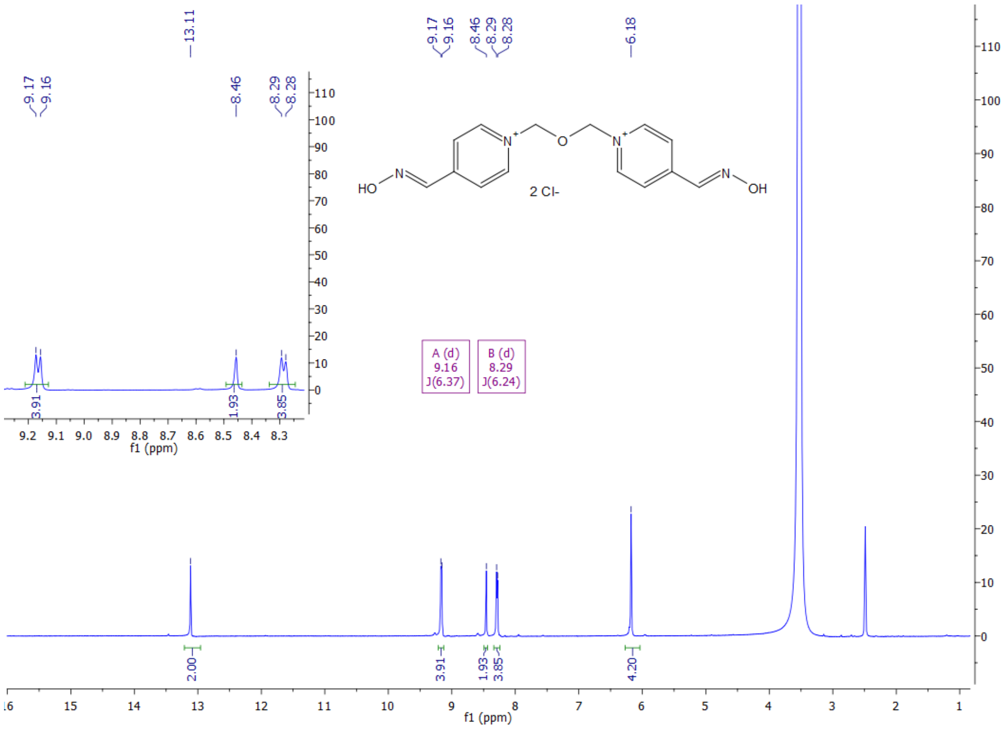

^{13}C NMR of obidoxime

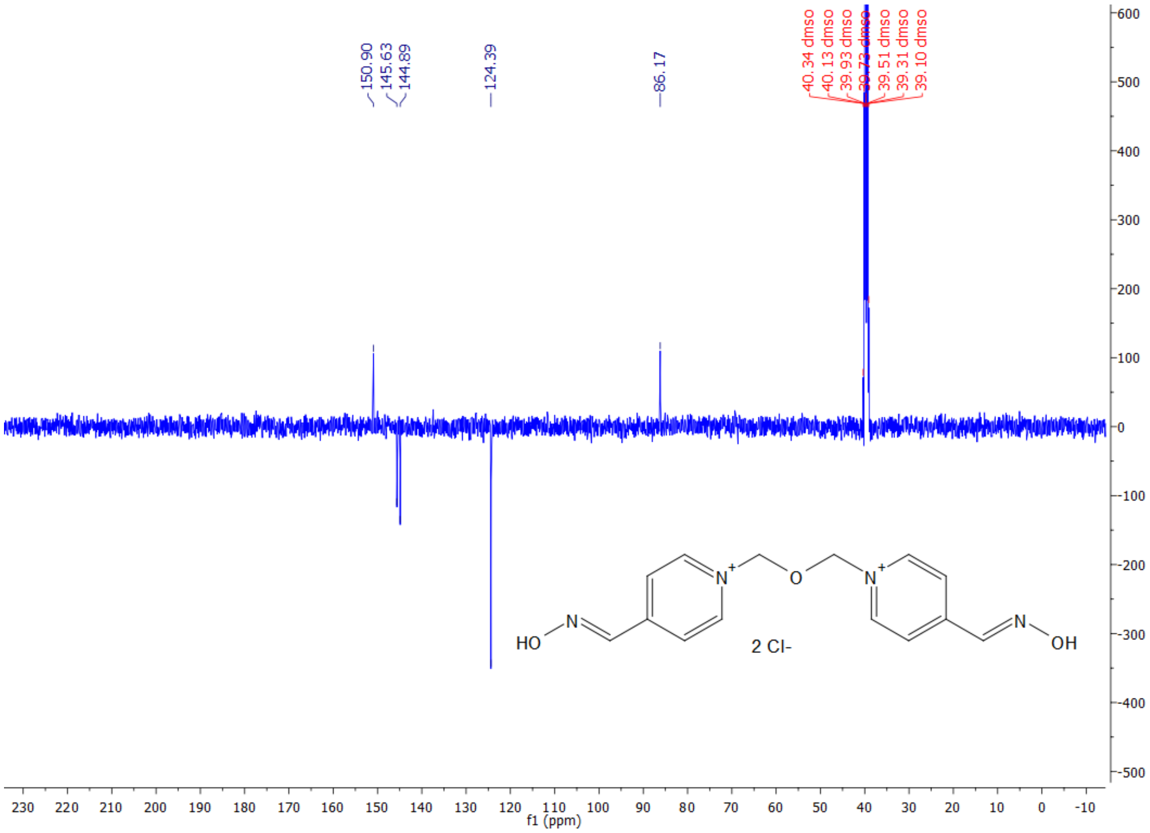

Supplement: Supplementary file 1 [file molecules-23-02954-s001.pdf]
